# Miscellaneous

**Published:** 1887-10

**Authors:** 


### MISCELLANEOUS.

The Crystal Pepsin Tablets, prepared by Carl L. Jensen, and advertised in this Journal, have acquired a wide-spread reputation for their curative properties in the treatment of dyspepsia and pharyngeal catarrh, This preparation is recognized in Germany as the most reliable of its kind attainable, and very many important cures, through its agency, are certified to in this and other countries. Give it a fair trial.

A most remarkable imitation of black walnut has lately been manufactured from poor pine, the quality and appearance of the article being such as to defy detection, except upon very close examination. To accomplish this, one part of walnut peel extract is mixed with six parts of water, and with this solution the wood is coated. When the material is half dry, a solution of bichromate of potash with water, is rubbed on t, and the made walnut is ready for use.

How She Understood It.—Maxie was the little six year old daughter of a clergyman, who had taken great pains with her religious instruction, and had held before her the goodness of the Supreme Being, so that she should have in her mind always His kindness and mercy as well as power. One morning, her mother passing the open door of the room in which the child was playing, saw Miss Maxie standing on a chair before the mirror, with her face close to it, scrutinizing her little phiz with great earnestness, and with a long sigh she remarked, "I *don't* see how God could have given me such a nose, when he *knows* how particular I am.—*Harper's Magazine.*

*Harper's Bazaar* has the following on the "Mind Cure:" "It appears to us a hopeful sign of a more wholesome life that large classes of women take time and spend money to hear this theory of the reality of a spiritual existence, expounded. After the poet, they are discovering that 'soul is form, and doth the body make.' They are semi-invalids. They have suffered. They have allowed their thoughts to dwell upon their pains and limitations, until these have come largely to fill their mental horizon. Their talk is of sickness. Even young girls, among the well-to-do and idle classes, compare symptoms and suggest diagnoses as staple topics of conversation. Nothing could be worse, it seems to us, on the score of good taste or of good health. As dwelling upon one's griefs magnifies them, so dwelling upon one's pains magnifies them. If the mind cure can be made to work upon these morbid subjects, it must restore to activity, energies worse than wasted; it would save time and money; it would make a grave world into a joyous one; it would multiply past calculations the sum of human happiness."

Winchester's Hypophosphite of Lime and Soda has gained a wide spread reputation for efficacy in the treatment of pulmonary diseases. We cordially recommend it to such of our readers as suffer from any phase of lung disorders. It may be procured of almost any leading druggist in any of our larger towns.

WATER AS A MEDICINE.—Ordinary drinking water, if taken in large quantities, acts as a solvent and a diuretic, and also increases the perspiration if the temperature of the air be high. Taken in the quantity of one or two quarts at a time, the diluent effect of water is often sufficient to eliminate an excess of alcohol from the blood, as after taking too much wine. Another effect of large draughts of water is to make the pulse slower, and to diminish slightly the normal temperature of the body.

Increase of weight has been claimed as a result of systematic water drinking on retiring for the night. The latest researches do not bear out this conclusion. Water thus taken will prevent any actual loss of weight. but it is not shown that it will do anything more. With the addition of a moderate stimulant, however, it has often a decidedly fattening effect. —*Harper's Magazine*

INFANT LOGIC.—"Mamma" said little Bobby, "can't I have another piece of pie?" "No, my dear. You have had sufficient." "But why can't I have more?" "It might make you sick, and if you become sick you might die." "Die just like Johnny Brown?" "Yes, my dear." "Everybody said he went to Heaven, mamma." "Yes, dear. He was a good little boy and always minded what his mamma told him." "And people said, mamma, that he'd be ever so much happier in Heaven than here." "That is right, Bobby. So he would." "Do all good little boys go to Heaven, mamma?" Yes, dear." "Am I a good little boy, mamma?" "Yes; Bobby is a good little boy." "Then why don't you let me have another piece of pie? Don't you want your little Bobby to be happy?"—*Pittsburgh Dispatch.*

### OH!

Clarissa was through with her boarding school,
What she did'nt know—but never mind that,
She knew what was meant by the golden rule,
And so did her John in the bower where they sat.

"Do unto others as you'd be done by,"
She said, in a voice deliciously low,
That drew out of John an answering sigh,
And then she said something that sounded like oh!

They'll be married next winter, the gossips all say,
The old folks have graciously come to agree.
They'll learn, too, that life is not all a play,
Perhaps they'll be happy! we'll just wait and see.

